# Clinical relevance of endoscopic peri-appendiceal red patch in ulcerative colitis patients

**DOI:** 10.1177/17562848221098849

**Published:** 2022-06-28

**Authors:** Maud A. Reijntjes, Lianne Heuthorst, Krisztina Gecse, Aart Mookhoek, Willem A. Bemelman, Christianne J. Buskens

**Affiliations:** Department of Surgery, Amsterdam UMC, University of Amsterdam, Amsterdam, The Netherlands; Department of Surgery, Amsterdam UMC, University of Amsterdam, Amsterdam, The Netherlands; Department of Gastroenterology, Amsterdam UMC, University of Amsterdam, Amsterdam, The Netherlands; Institute of Pathology, University of Bern, Bern, Switzerland; Department of Surgery, Amsterdam UMC, University of Amsterdam, Amsterdam, The Netherlands; IBD Unit, Gastroenterology and Endoscopy, IRCCS Ospedale San Raffaele and Vita-Salute San Raffaele University, Milano, Italy; Surgeon, J1A-216, Amsterdam UMC, location AMC, Meibergdreef 9, Amsterdam 1105 AZ, The Netherlands

**Keywords:** appendix, endoscopy, inflammation, ulcerative colitis

## Abstract

**Background:**

Increasing evidence is suggesting appendectomy as an alternative treatment for ulcerative colitis (UC), especially in case of histological appendiceal inflammation. Therefore, preoperative identification of appendiceal inflammation could be beneficial. This study aimed to assess the prevalence of peri-appendiceal red patch (PARP) on colonoscopy. In addition, prognostic relevance of PARP for disease course and its predictive value for histological appendiceal inflammation in patients undergoing appendectomy was assessed.

**Methods:**

UC patients undergoing colonoscopy in 2014/2015 were included to determine PARP-prevalence in a cross-sectional study. Findings were correlated to patient and disease characteristics, upscaling of treatment and colectomy rates after cross-sectional colonoscopy. In patients undergoing appendiceal resection, histopathological inflammation was assessed using the Robarts Histopathology Index (RHI).

**Results:**

In total, 249 patients were included of which 17.7% (44/249) had a PARP. Patients with PARP were significantly younger with a shorter disease course. The majority of patients with PARP (61.4%) was in endoscopic remission. Patients with PARP required more upscaling of medical therapy (81.8% *vs*. 58.0%, *p* < 0.01), and more PARP patients underwent colectomy (13.6% *vs*. 4.9%, *p* = 0.04). Patients with PARP had a higher median RHI in resection specimens (14 *vs*. 7, *p* < 0.01).

**Conclusion:**

PARP was present during colonoscopy regardless disease activity and was predominantly found in UC patients with younger age and shorter disease duration. PARP patients had a more severe course of UC, and in case of appendectomy, more severe histopathological appendiceal inflammation. Appendectomy as an experimental therapy for UC has been suggested to be predominantly effective in UC patients with appendiceal inflammation. This study demonstrates that presence of a PARP on colonoscopy predicts appendiceal inflammation. After consensus has been reached on the therapeutic effect of appendectomy, assessing PARP presence during colonoscopy could therefore contribute to identifying patients most likely to respond.

## Introduction

In the past decades, evidence has been accumulating linking the appendix to immunologic functions in the pathogenesis of ulcerative colitis (UC).^1^ Patients who underwent appendectomy during childhood have a lower risk for developing UC.^2,3^ For this reason, surgeons have been performing experimental therapeutic removal of the appendix in patients with UC.^4,5^ By performing appendectomy, physicians strive to mitigate disease activity, reduce relapses, and eventually postpone or prevent colectomy. Two prospective studies have demonstrated that in patients with (therapy refractory) UC undergoing appendectomy, clinical response ranges from 46% to 90%, with 25% of patients remaining in endoscopic remission up to over 4 years after appendectomy.^4,5^ Histopathological inflammation has been found in the majority of appendiceal resection specimens (range 44–80%) of UC patients.^6–8^ Recent studies demonstrated that UC patients with active appendiceal inflammation (ulcerative appendicitis) were more likely to benefit from appendectomy.^9,10^ When aiming for a patient-tailored treatment approach, preoperative identification of appendiceal inflammation could therefore be a clinically relevant step.

Although intraluminal endoscopic assessment of the appendix is impossible due to its narrow lumen, inflammation of the appendiceal orifice or ‘mouth’ has frequently been described during endoscopy in UC patients. This phenomenon is called ‘peri-appendiceal red patch’ (PARP), ‘peri-appendiceal inflammation (PAI)’ or ‘cecal patch’ and has typical endoscopic features of UC, such as mucosal erythema, ulceration, fibrin deposit or mucosal bleeding. The prevalence of PARP in UC patients varies from 8% to 75% depending on definition and inclusion criteria of studies.^11^ It has been predominantly described in distal colitis; proctitis in particular.^12,13^ Previous studies show conflicting results regarding the clinical significance of a PARP on the course of UC.^12,14–17^

The aim of this study was to assess the prevalence of PARP on colonoscopy in a cross-sectional UC cohort, and correlate findings to patient and disease characteristics. In addition, the prognostic relevance of a PARP was analyzed by comparing upscaling of medical treatment and colectomy rates during follow-up after cross-sectional colonoscopy. Finally, the relation between PARP and histopathological findings of the appendix in resection specimens of patients undergoing appendectomy or colectomy was analyzed to determine if the endoscopic finding of PARP is correlated to appendiceal inflammation.

## Materials and methods

### Study design

After institutional review board approval, all consecutive patients with a (new) diagnosis of UC according to ECCO guidelines who underwent a colonoscopy between 1 January, 2014 and 1 January, 2016 at the Amsterdam University Medical Center, location AMC were included in this retrospective cohort study.^18^ Patients of ⩾18 years old who underwent a colonoscopy with intubation of the cecum were included. Patients with previous colonic resection or appendectomy, a defunctioned colon, or with a suspicion of Crohn’s disease were excluded. The reporting of this study conforms to the STROBE statement.^19^

### Endoscopic assessment

Assessment and documentation of the appendiceal orifice during colonoscopy performed by a gastroenterologist was standard practice in the Amsterdam University Medical Center, location AMC. Two investigators (MR and LH) extracted patient and endoscopic data including images, which was followed by a central reinvestigation (MR) assessing PARP presence in the entire study cohort blinded for prior assessment. A PARP was scored positive if a PARP was described in the endoscopy report and/or a PARP was present on images of the appendiceal orifice taken by the gastroenterologist during the endoscopic procedure. Images were reassessed and scored by a colorectal surgeon (CB) and/or gastroenterologist (KG) if there was disagreement between the report and images or between researchers. PARP was scored negative if one of the following situations applied:

1. The endoscopy report did not describe the appendiceal orifice but images showed a normal appendiceal orifice.
2. The endoscopy report described a normal appendiceal orifice in absence of corresponding images.
3. The endoscopy report described absence of disease in entire colon in absence of corresponding images.
4. The endoscopy report and/or images described continuous cecal disease activity including the appendiceal orifice, since identification of PARP as a skip lesion could be precluded.

If multiple endoscopies on one patient were performed during 2014 and 2015, the colonoscopy with the highest Boston Bowel Preparation Score for fecal contamination was included.^20^ Patients who underwent colonoscopy in 2014 or 2015 were included to assess disease prognosis with a sufficient follow-up period.

### Outcome measures

The prevalence of a PARP was determined in this cross-sectional UC population. Of the included patients, endoscopy reports before January 2014 or after December 2015 in the AMC were also assessed for (previous) presence of a PARP, which were included in the longitudinal analyses. Follow-up was defined as number of months between cross-sectional colonoscopy in 2014/2015 and either last hospital visit date or colectomy.

#### Definition of outcome variables

##### Patient demographics

Data on patient characteristics at time of cross-sectional colonoscopy (i.e. gender, age, medication) and disease characteristics at time of cross-sectional colonoscopy (disease onset, clinical symptoms, colonoscopy indication) were extracted retrospectively from electronic patient files. Data on disease location and endoscopic Mayo scores (EMS) were directly extracted from the corresponding colonoscopy report.^21^

##### Endoscopic disease activity

Disease activity was defined as remission in case of an EMS of ⩽1 *versus* active disease in case of an EMS ⩾ 2. Disease extension was defined as a minimum of 10 cm proximal disease migration on follow-up endoscopy.

##### Upscaling of treatment

Upscaling of therapy during follow-up after cross-sectional colonoscopy was defined by either increasing dose or frequency of existing medication and/or adding medication as therapeutic policy. An upscale should be based on suspicion for a flare based on clinical and/or biochemical (calprotectin and C-reactive protein) and/or endoscopic disease activity. Restart of stopped medical therapy was also considered an upscale of medication.

##### Colectomy

Both (sub)total colectomy and segmental resection of colonic tissue (as treatment for colonic malignancy) during follow-up were included as events in survival analyses.

### Pathological assessment

In patients who underwent appendectomy or colectomy during follow-up, appendiceal specimens were re-evaluated histopathologically. A gastrointestinal pathologist specialized in IBD (AM) centrally scored hematoxylin and eosin stained slides of appendiceal specimens using the Robarts Histopathology Index (RHI).^22^ The RHI includes four categories that are scored from 0 to 3 (Supplementary file 1). The RHI score ranges between 0 (no disease activity) and 33 (severe disease activity).

### Statistical analyses

The prevalence of a PARP was assessed using descriptive statistics. Patient characteristics, disease characteristics, colectomy rates, and the RHI score of appendiceal resection specimens were compared between patients with a PARP and without a PARP. Categorical data were presented as frequencies and percentages. To compare dichotomous data, the Chi-square test or Fisher’s exact test were used, as appropriate. Continuous data were presented as mean and standard deviation (SD) or as median and interquartile range (IQR), according to their distribution. To compare continuous data, The Mann–Whitney *U* test was used for not normally distributed data. Normally distributed continues data were analyzed with Student’s *t* test. Follow-up data were calculated starting from the date of (ileo)colonoscopy until the last date of outpatient visit. Survival data were described in months, survival analyses were displayed by Kaplan–Meier curves and the Log-rank test was used to compare data. All tests were two-sided, with a level of significance set a *p* < 0.05. Statistical analyses were performed using SPSS (IBM Corp., Armonk, NY, USA).

## Ethical considerations

This study was waived from review of the medical ethics boards of the Amsterdam UMC, location AMC in December 2019, since the data collection did not interfere with the integrity of the patients. All patients were asked study participation and informed consent by opt-out letter. Patients who declined study participation during the opt-out procedure were excluded.

## Results

**Figure 1.**
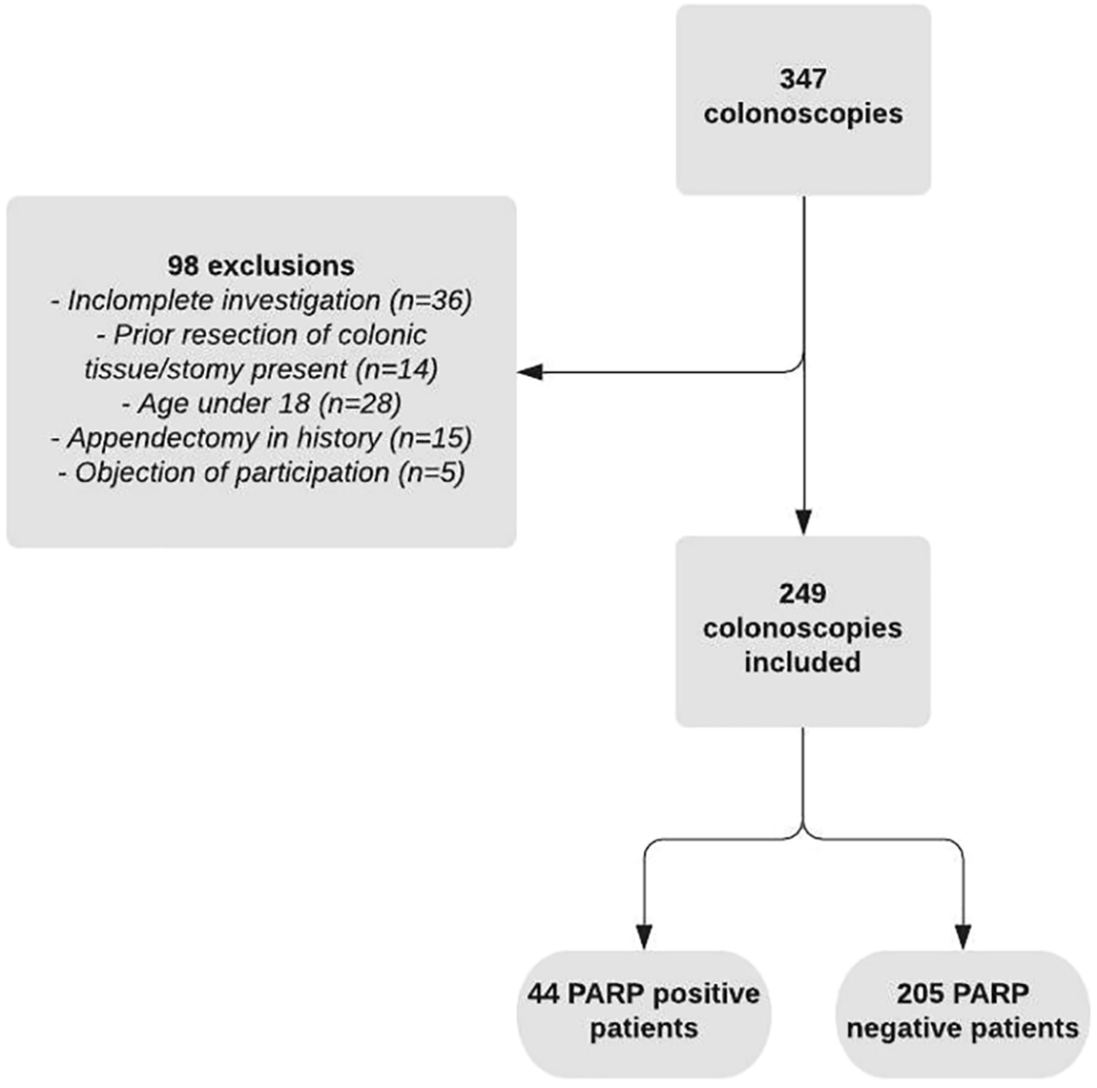
Flowchart of patient inclusion.

A total of 347 consecutive patients with UC underwent a (ileo)colonoscopy, of which 98 were excluded. Most investigations (36/98) were excluded due to incomplete investigation (e.g. inability to intubate cecal base, severe fecal contamination), age under 18 (28/98) or previous appendectomy (15/98). Other reasons for exclusion are displayed in Figure 1. The majority of (ileo)colonoscopies (145/249 = 58.2%) was performed for dysplasia surveillance. Other indications were efficacy assessment of a therapeutic intervention (50/249 = 20.1%), suspicion of exacerbation (41/249 = 16.5%), or initial UC diagnosis (13/249 = 5.2%). Out of 249 patients, 134 were male (53.8%). The median age at time of colonoscopy was 48.0 (IQR 35.0–61.0) years, with a median disease duration of 13.0 (17.0–24.0) years. The median follow-up for this cohort was 66 (IQR 56.0–74.0) months. Remaining patient characteristics are displayed in the baseline table (Table 1).

**Table 1.** Baseline characteristics.

Patient characteristics	Total (*n* = 249)		PARP (*n* = 44)		No PARP (*n* = 205)			Missing	
	*n*	%	*n*	%	*n*	%	*p* value	*n*	%
Gender									
Male	134	53.8%	21	47.7%	113	55.1%	0.37	0	
Age at time of scopy. years. median(IQR)	48.0 (35.0–61.0)		41.0 (32.3–50.8)		50.0 (36.0–63.0)		**<0.01**	0	
Disease duration. years. median(IQR)	13.0 (17.0–24.0)		9.5 (3.0–24.3)		14.5 (7.8–24.3)		**0.05**	5	2.0%
Active disease location (*n* = 69)									
Proctitis	10	14.5%	4	23.5%	6	11.5%	0.51	0	
Left sided	25	36.2%	5	29.4%	20	38.5%			
Extended	34	49.3%	8	47.1%	23	44.2%			
Endoscopic mayo score									
0 or 1	180	72.3%	27	61.4%	153	74.6%	0.07	0	
2 or 3	69	27.7%	17	38.6%	52	25.4%			
Colonoscopy indication									
Initial UC diagnosis	13	5.2%	4	9.1%	9	4.4%	**0.02**		
Surveillance	145	58.2%	18	40.9%	127	62.0%			
Suspicion of exacerbation	41	16.5%	7	15.9%	34	16.6%			
Assess effect therapy	50	20.1%	15	34.1%	35	17.1%			
PSC/IBD	38	15.3%	5	11.4%	33	16.1%	0.30	35	14.1%
Medication at time of scopy									
None	43	17.3%	8	18.2%	35	17.1%	0.09	1	0.4%
5-asa	103	41.4%	13	29.5%	90	43.9%			
Immunosuppressants	59	23.7%	10	22.7%	49	23.9%			
Biologicals	43	17.3%	13	29.5%	30	14.6%			

IQR: interquartile range; PARP: peri-appendiceal red patch; UC: ulcerative colitis; PSC: primary sclerosing cholangitis; IBD: inflammatory bowel disease; PSC/IBD: Concurrent diagnosis of primary sclerosing cholangitis and inflammatory bowel disease.

a Interquartile range.

b Oral/systemic steroids included.

c Trial medication included.

### PARP presence

**Figure 2.**
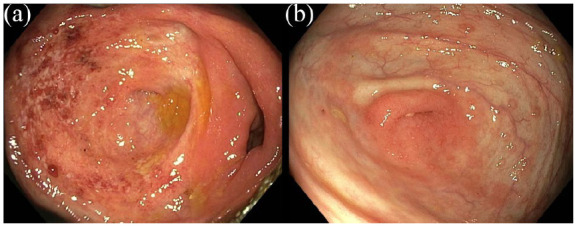
(a, b) Peri-appendiceal inflammation on colonoscopy.

Screenshot images of the appendiceal orifice during colonoscopy were present in 163 out of 249 (65%) patients, and in 86 (35%) patients colonoscopy reports mentioned the appendiceal orifice. Forty-four out of 249 (17.7%) colonoscopies showed a PARP as a skip lesion on colonoscopy (Figure 2(a) and (b)), all of which were visible on corresponding screenshot images. A mild PARP (erythema, lack of vascular pattern) was found in 33 patients; eleven patients demonstrated a severe PARP (ulcerations, bleeding). A higher incidence of PARP was found in patients undergoing colonoscopy investigation for initial diagnosis of UC, assessment of therapy efficacy and suspicion of UC exacerbation (*n* = 4, 30.8% and *n* = 15, 30%, and *n* = 7, 17.1%, respectively), when compared to planned dysplasia surveillance colonoscopy investigations (*n* = 18, 12.4%, *p* = 0.02). If 20 patients with continuous cecal inflammation including the appendiceal orifice would have been added to the PARP-group, the prevalence of PARP would increase to 25.7% (64/249). As continuous inflammation of the caecum precluded identification of a PARP, these patients were allocated to the PARP-negative group during further analyses. Analyzing all colonoscopies of included patients performed before January 2014 or after January 2016, 22 additional patients were demonstrated to have (had) a PARP, resulting in a longitudinal incidence of 66/249 (26.5%) in this UC cohort; 12 patients in history and 10 during follow-up.

### Patient demographics

Mean age of disease onset and gender were not significantly different in patients with and without PARP (Table 1). Patients with PARP were significantly younger (42.3 *vs* 49.3 years, *p* < 0.01), and had a shorter disease duration when compared with patients without PARP (9.5 *vs* 14.5 years, *p* = 0.05). The majority of patients with PARP was in endoscopic remission (61.4%), as compared with 72.3% in the entire study cohort. Numerically, patients with PARP were more frequently demonstrated to have proctitis (23.5% *vs* 11.5%) and more frequently used biologicals or trial medication (29.5% *vs* 14.6%) when compared with the PARP-negative group, although differences were not statistically significant.

### Prognostic significance of PARP

#### Upscaling of medical treatment

**Figure 3.**
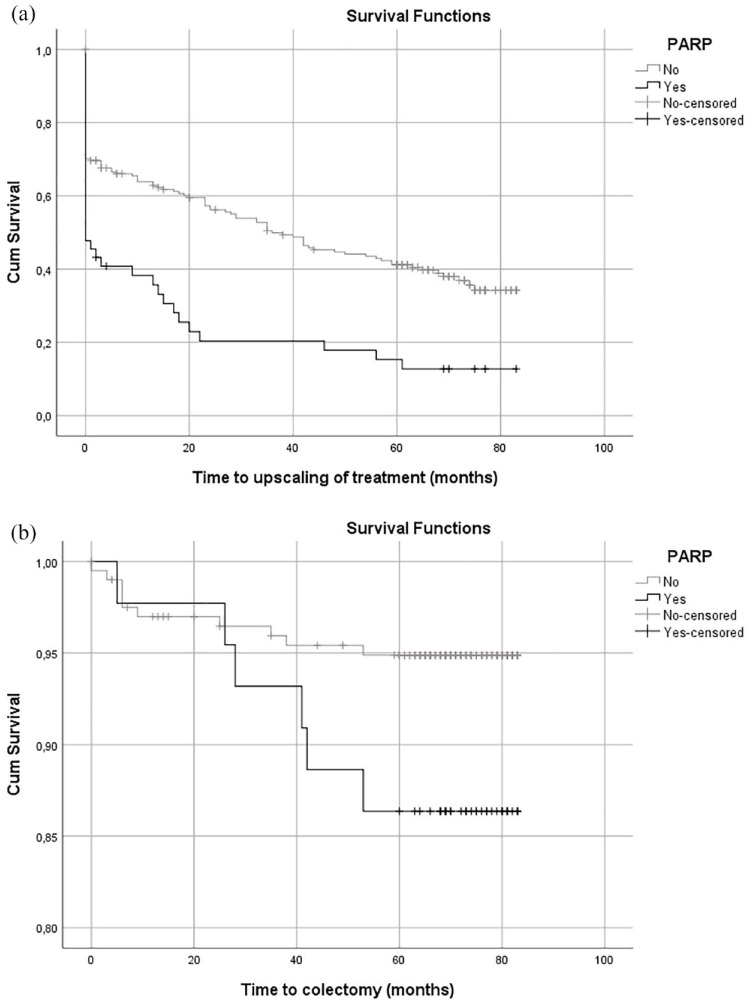
Outcomes of disease activity during follow-up after cross-sectional colonoscopy: (a) upscaling of (medical) treatment and (b) colectomy.

Four out of 249 patients (1.6%) were lost to follow-up and excluded from prognostic analyses. During follow-up, 156 patients (63.9%) required upscaling of medical treatment. This proportion was significantly higher for 37 out of 44 patients with PARP, when compared with 119 out of 201 patients without PARP (84.1% *vs* 59.2%, *p* < 0.01). Moreover, PARP-positive patients had a shorter median time to upscaling of (medical) treatment compared with PARP-negative patients (0.0 *vs* 36.0 months, *p* < 0.01, Figure 3(a)). A subanalyses on the patient cohort who underwent a planned colonoscopy for dysplasia surveillance (*n* = 145) demonstrated a similar decrease in median time to upscaling of (medical) therapy outcomes for PARP-positive patients (15.0 *vs* 69.0 months, *p* = 0.01).

#### Colectomy

The overall colonic resection rate was 6.5% (16/245). Seven patients underwent a (sub)total colectomy for therapy refractory disease. One patient underwent an abdominoperineal resection for a UC-related stenosis, and one patient developed severe diverticulosis for which a recto-sigmoid resection was performed. Seven UC patients developed colonic dysplasia or malignancy, of which one underwent a recto-sigmoid resection and six patients underwent a (sub)total colectomy. Out of 44 patients with PARP, six underwent a colonic resection, compared with 10/201 of patients without PARP (13.6% *vs* 5.0%, *p* = 0.05). Colonic resection rates for therapy refractory UC were also higher for PARP-positive patients when compared with PARP-negative patients (both *n* = 4, 9.1% *vs* 2.0%, *p* = 0.04). PARP was not involved in the therapeutic decision making regarding resection in any of the patients. Figure 3(b) shows the survival curve for colonic resection for patients with and without PARP.

#### Proximal disease extension

Proximal disease extension on endoscopy occurred in 12 out of 35 (34.3%) patients with proctitis or left-sided disease. A higher proportion of patients with PARP developed proximal disease extension when compared to patients without PARP (6/9, 66.7% *vs* 6/26, 23.1%), although this difference was not significant (*p* = 0.06)

### Histopathological inflammation of appendices

A total of 31 patients underwent resection of the appendix or colectomy during follow-up. Twenty-three appendiceal resection specimens were available for histopathological inflammation (RHI) assessment. Seven resection specimens could not be scored as patients were operated in another hospital without the possibility to retrieve slides. One appendix specimen could not be evaluated due to the condition of the slides.

**Figure 4.**
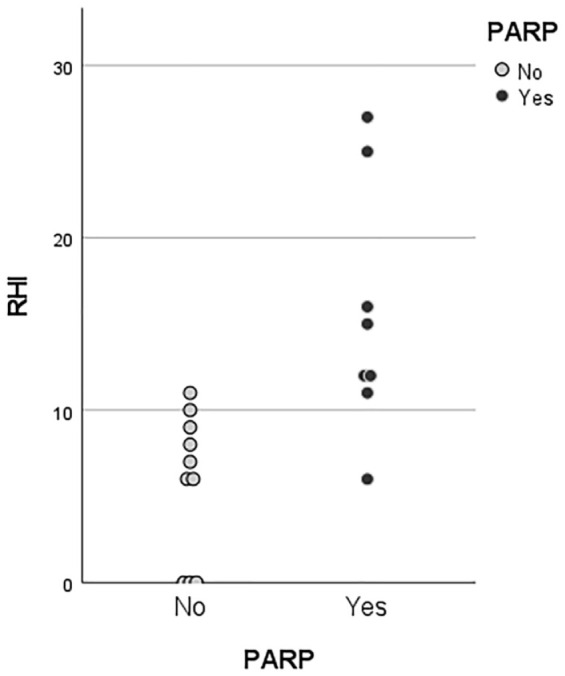
RHI scores of resected appendices with (black) and without (gray) PARP.

Five patients had a complete fibrotic appendix on histology, which precludes scoring the RHI. One patient with PARP developed acute appendicitis treated with appendectomy. The overall median RHI score of the remaining appendiceal specimens was 10 (IQR 6–13). Patients with PARP (*n* = 8) scored a higher median Robarts histopathological index (14, IQR 0–9) when compared with patients (*n* = 10) without PARP (7, IQR 11–23, *p* < 0.01, Figure 4).

## Discussion

In this cross-sectional cohort study, a PARP prevalence of 17.7% was found in patients with UC. PARP was predominantly found in younger patients with shorter disease duration, corresponding with available literature.^23,24^ It was demonstrated that this endoscopic finding had prognostic relevance with more frequent and earlier upscaling of medical treatment and higher colectomy rates in general and for refractory UC in patients with PARP. The prevalence of PARP in this study was relatively low, although well within range as previously described in the systematic review by Park *et al.*^11^ (8–75%). This wide range is probably due to heterogeneous study cohorts when it comes to inclusion criteria, disease location and definition of PARP. Some studies showing high PARP prevalence only included patients with proctitis,^12,13,16,25^ corresponding with results of this study demonstrating a PARP was more frequently found in patients with active proctitis (29.4%). Besides, other studies evaluating PARP presence either excluded colonoscopies describing cecal disease or defined patients with cecal inflammation PARP positive.^13,23,26,27^ However, it is difficult to reliably discriminate inflammation around the appendix orifice from continuous inflammation in caecum. In this study, absence of PARP was presumed in presence of cecal disease. Including these patients in the PARP-positive group would increase the prevalence to 25.7%. With a median disease duration of 13 years and a follow-up of 66 months, the longitudinal incidence of PARP increased up to 27% in this study.

In the current series, only 5% of patients who were initially diagnosed with UC on colonoscopy had an endoscopic assessment without the influence of (previous) medication. However, no relation between medication use and PARP presence was found, which corresponds to published studies.^28^ The cohort of patients undergoing colonoscopy for medical therapy assessment did not demonstrate a significant increase of PARP incidence, as the highest incidence of PARP (30.8%) was found in patients who underwent colonoscopy with initial UC diagnosis as outcome. This finding corresponds with the hypothesis that appendiceal inflammation occurs in an early disease stage of UC as it has a role in UC disease etiology. The majority of patients in this cohort were in endoscopic remission as a large proportion of patients undergoing colonoscopy had surveillance (58.2%) as indication. Patients undergoing surveillance colonoscopy demonstrated to have a low incidence of PARP (12.4%). However, the prognostic impact of PARP remained significant for the surveillance cohort when it comes to upscaling of medical treatment. Obviously, performing a colonoscopy for dysplasia surveillance might result in less focus on PARP presence. Although this theoretically could have caused an underestimation of PARP prevalence, documentation, and photography of the appendix orifice was standard procedure during endoscopy and an independent re-evaluation of colonoscopy images and reports was additionally performed. A lower proportion of patients underwent a colonoscopy for suspected disease activity, as sigmoidoscopy generally suffices for these patients.

Endoscopic remission (EMS ⩽ 1) was found in 61.4% of colonoscopies demonstrating PARP. This finding suggests that despite the absence of colonic disease activity, appendiceal inflammation can in fact be present, corresponding with available literature.^23,27^ Retrospective studies have suggested that an appendectomy in UC patients in remission might result in decreased relapses.^29^ It is interesting to speculate whether this could be related to the finding of ongoing inflammation in the appendix.

So far, literature on prognostic significance of PARP presence shows conflicting results.^14–17^ The largest study demonstrated that the presence of PARP has prognostic implication in the disease course of UC including remission, relapse and proximal disease extension.^11^ This study confirms the prognostic relevance of PARP as it could be demonstrated that both upscaling of medical treatment and colectomy rates were higher in PARP-positive patients. Again, this finding is intriguing in light of the previous findings in literature that an appendectomy in UC has been associated with reduced relapses, mitigation of disease activity and the prevention (or postponing) colectomy.^4,9^

Segmental colectomy for UC-related dysplasia or cancer may intuitively appear as an inconsequent strategy as guidelines suggest to consider procotocolectomy. However, an increasing number of reports suggest that segmental colectomy could represent an alternative in selected (older) UC patients without active colitis.^30,31^ The rate of patients who underwent segmental colectomy for UC-associated dysplasia or cancer in the current study cohort (1 out of 7 patients) is lower when compared with results of a recently published population-based study. This Canadian study reported that 46% of UC patients with dysplasia or cancer underwent segmental resection between 2007 and 2015.^30^ The expertise of PSC/IBD in our tertiary center resulted in a relatively high incidence of PSC in this UC study cohort. PSC/IBD patients demonstrated to have numerically lower incidence of PARP in the current study. As right-sided disease is a classical disease location of PSC/IBD patients, our interpretation of PARP absence in case of right-sided (cecal) disease could explain this finding.^32^

According to multiple studies, patients with a histologically inflamed appendix appear to benefit more from experimental therapeutic appendectomy in UC.^9,10^ Prospective trials should establish the therapeutic role of appendectomy and the association between histopathological inflammation and response to therapeutic appendectomy.^33^ In due course, preoperative identification of appendiceal inflammation by endoscopic PARP may contribute to improved patient-tailored treatment selection in the ever-growing treatment armamentarium. Studies found higher appendiceal histological inflammation rates in PARP-positive patients before when compared with the proportion in this study. Most of these studies, however, assessed biopsies of the appendiceal orifice or colectomy specimens.^11,23,27^ This study showed significantly higher RHI scores in PARP patients compared with PARP-negative patients, assessing both appendix and colectomy resection specimens.

One of the limitations of this study is the retrospective and heterogeneous nature of the study cohort, which challenges accurate assessment of the significance of PARP. The advantage of including all patients in a consecutive series, however, results in a more reliable estimation of the true PARP prevalence in UC patients in one of the largest studies conducted on this topic. In addition, it can be hypothesized that the endoscopic finding of a PARP was part of the indication for upscaling therapy, which could influence the prognostic relevance of results. However, PARP is generally considered as a skip lesion, and there is no established indication for therapy upscaling solely based on this finding.

Another limitation is the relatively small cohort and number of events in prognostic assessment. Although differences on colectomy rates between groups are significant, the amount of patients undergoing colectomy is relatively low with only eight colectomies being performed for therapy refractory UC during follow-up. However, we decided to include patients undergoing colectomy with malignancy as indication as the development of a malignancy in a UC patient is often related to long-standing disease activity. Moreover, despite low numbers of appendiceal resection specimens, the increased appendiceal inflammation for PARP-positive patients was remarkable with only two patients having a mildly inflamed appendix in the PARP-negative group.

In conclusion, 17.7% of UC patients showed a PARP during colonoscopy performed in 2014 or 2015. PARP presence was higher in younger patients with shorter disease duration. The majority of PARP-positive patients was in endoscopic remission. Rates for colectomy- and upscaling of treatment were higher for patients with PARP. PARP-positive patients demonstrated a higher RHI score. Since patients with histopathological inflammation of the appendix tend to respond better to experimental therapeutic appendectomy, endoscopic identification of a PARP could contribute to identifying patients most likely to benefit from appendectomy.

## Plain Language Summary

**Clinical relevance of endoscopic peri-appendiceal red patch in ulcerative colitis patients**

Increasing evidence is suggesting surgical removal of the appendix vermiformis as an alternative treatment for ulcerative colitis (UC), especially in case of histopathological inflammation of the appendix. Preoperative assessment of appendiceal inflammation could therefore facilitate identifying UC patients suitable for appendectomy. Presence of peri-appendiceal red patch (PARP) on colonoscopy and its prognostic value was assessed in this retrospective study. We found a PARP on colonoscopy in 44/249 (18%) of patients. UC patients with a PARP require upscaling of medical therapy and colectomy more frequently during follow-up. The majority of patients with PARP (61%) had a maximum endoscopic Mayo score of 1 in the remaining colon on endoscopy, and thus had quiescent UC disease. Patients with PARP had a more severely inflamed appendiceal resection specimen after appendiceal resection, according to the measured Robarts Histopathology Index. As patients with a histologically inflamed appendix respond better to therapeutic appendectomy, this study emphasizes the relevance of preoperative assessment of PARP presence on colonoscopy in UC patients.

## Supplemental Material

sj-docx-1-tag-10.1177_17562848221098849 – Supplemental material for Clinical relevance of endoscopic peri-appendiceal red patch in ulcerative colitis patients Supplemental material, sj-docx-1-tag-10.1177_17562848221098849 for Clinical relevance of endoscopic peri-appendiceal red patch in ulcerative colitis patients by Maud A. Reijntjes, Lianne Heuthorst, Krisztina Gecse, Aart Mookhoek, Willem A. Bemelman and Christianne J. Buskens in Therapeutic Advances in Gastroenterology

## References

[1] Sahami S Kooij IA Meijer SL , et al. The link between the appendix and ulcerative colitis: clinical relevance and potential immunological mechanisms. Am J Gastroenterol 2016; 111: 163–169. 26416189 10.1038/ajg.2015.301

[2] Rutgeerts P D’Haens G Hiele M , et al. Appendectomy protects against ulcerative colitis. Gastroenterology 1994; 106: 1251–1253. 8174886 10.1016/0016-5085(94)90016-7

[3] Andersson RE Olaison G Tysk C , et al. Appendectomy and protection against ulcerative colitis. N Engl J Med 2001; 344: 808–814. 11248156 10.1056/NEJM200103153441104

[4] Stellingwerf ME Sahami S Winter DC , et al. Prospective cohort study of appendicectomy for treatment of therapy-refractory ulcerative colitis. Br J Surg 2019; 106: 1697–1704. 31393608 10.1002/bjs.11259

[5] Bolin TD Wong S Crouch R , et al. Appendicectomy as a therapy for ulcerative proctitis. Am J Gastroenterol 2009; 104: 2476–2482. 19584834 10.1038/ajg.2009.388

[6] Jo Y Matsumoto T Yada S , et al. Histological and immunological features of appendix in patients with ulcerative colitis. Dig Dis Sci 2003; 48: 99–108. 12645797 10.1023/a:1021742616794

[7] Scott IS Sheaff M Coumbe A , et al. Appendiceal inflammation in ulcerative colitis. Histopathology 1998; 33: 168–173. 9762551 10.1046/j.1365-2559.1998.00477.x

[8] Sahami SGT Gardenbroek TJ Straalen JP , et al. Lymphocytes populations in appendiceal lavage fluid predictive of IBD-related inflammation. Gastroenterol Hepatol Open Access 2018; 9: 65–72.

[9] Sahami S Wildenberg ME Koens L , et al. Appendectomy for therapy-refractory ulcerative colitis results in pathological improvement of colonic inflammation: short-term results of the PASSION study. J Crohns Colitis 2019; 13: 165–171. 30285094 10.1093/ecco-jcc/jjy127

[10] Heuthorst L Mookhoek A Wildenberg ME , et al. High prevalence of ulcerative appendicitis in patients with ulcerative colitis. United European Gastroenterol J 2021; 9: 1148–1156. 10.1002/ueg2.12171 PMC8672077 34750986

[11] Park SH Loftus EV Jr Yang SK. Appendiceal skip inflammation and ulcerative colitis. Dig Dis Sci 2014; 59: 2050–2057. 24705639 10.1007/s10620-014-3129-z

[12] D’Haens G Geboes K Peeters M , et al. Patchy cecal inflammation associated with distal ulcerative colitis: a prospective endoscopic study. Am J Gastroenterol 1997; 92: 1275–1279. 9260788

[13] Rubin DT Rothe JA. The peri-appendiceal red patch in ulcerative colitis: review of the University of Chicago experience. Dig Dis Sci 2010; 55: 3495–3501. 20936357 10.1007/s10620-010-1424-x

[14] Bakman Y Katz J Shepela C. Clinical significance of isolated peri-appendiceal lesions in patients with left sided ulcerative colitis. Gastroenterology Res 2011; 4: 58–63. 27942315 10.4021/gr302w PMC5139755

[15] Anzai H Hata K Kishikawa J , et al. Appendiceal orifice inflammation is associated with proximal extension of disease in patients with ulcerative colitis. Colorectal Dis 2016; 18: O278–O282. 27354363 10.1111/codi.13435

[16] Naves JE Lorenzo-Zúñiga V Marín L , et al. Long-term outcome of patients with distal ulcerative colitis and inflammation of the appendiceal orifice. J Gastrointestin Liver Dis 2011; 20: 355–358. 22187699

[17] Byeon JS Yang SK Myung SJ , et al. Clinical course of distal ulcerative colitis in relation to appendiceal orifice inflammation status. Inflamm Bowel Dis 2005; 11: 366–371. 15803026 10.1097/01.mib.0000164018.06538.6e

[18] Harbord M Eliakim R Bettenworth D , et al. Third European evidence-based consensus on diagnosis and management of ulcerative colitis. Part 2: Current Management. J Crohns Colitis 2017; 11: 769–784. 28513805 10.1093/ecco-jcc/jjx009

[19] von Elm E Altman DG Egger M , et al. The strengthening the reporting of observational studies in epidemiology (STROBE) statement: guidelines for reporting observational studies. Int J Surg 2014; 12: 1495–1499. 25046131 10.1016/j.ijsu.2014.07.013

[20] Lai EJ Calderwood AH Doros G , et al. The Boston bowel preparation scale: a valid and reliable instrument for colonoscopy-oriented research. Gastrointest Endosc 2009; 69: 620–625. 19136102 10.1016/j.gie.2008.05.057 PMC2763922

[21] Ket SN Palmer R Travis S. Endoscopic disease activity in inflammatory bowel disease. Curr Gastroenterol Rep 2015; 17: 50. 26650939 10.1007/s11894-015-0470-0 PMC4674531

[22] Mosli MH Feagan BG Zou G , et al. Development and validation of a histological index for UC. Gut 2017; 66: 50–58. 26475633 10.1136/gutjnl-2015-310393

[23] Matsumoto T Nakamura S Shimizu M , et al. Significance of appendiceal involvement in patients with ulcerative colitis. Gastrointest Endosc 2002; 55: 180–185. 11818919 10.1067/mge.2002.121335

[24] Horie H. Significance of periappendiceal inflammation in the patients with distal ulcerative colitis. Digestive Endoscopy 1999; 11: 119–124.

[25] Yamagishi N Iizuka B Nakamura T , et al. Clinical and colonoscopic investigation of skipped periappendiceal lesions in ulcerative colitis. Scand J Gastroenterol 2002; 37: 177–182. 11843054 10.1080/003655202753416849

[26] Takizawa. Appendiceal involvement in patients with ulcerative colitis. Digestive Endoscopy 2007; 9: 217–221.

[27] Ladefoged K Munck LK Jorgensen F , et al. Skip inflammation of the appendiceal orifice: a prospective endoscopic study. Scand J Gastroenterol 2005; 40: 1192–1196. 16265776 10.1080/00365520510023305

[28] Yang SK Jung HY Kang GH , et al. Appendiceal orifice inflammation as a skip lesion in ulcerative colitis: an analysis in relation to medical therapy and disease extent. Gastrointest Endosc 1999; 49: 743–747. 10343220 10.1016/s0016-5107(99)70293-2

[29] Naganuma M Iizuka B Torii A , et al. Appendectomy protects against the development of ulcerative colitis and reduces its recurrence: results of a multicenter case-controlled study in Japan. Am J Gastroenterol 2001; 96: 1123–1126. 11316158 10.1111/j.1572-0241.2001.03757.x

[30] Bogach J Pond G Eskicioglu C , et al. Extent of surgical resection in inflammatory bowel disease associated colorectal cancer: a population-based study. J Gastrointest Surg 2021; 25: 2610–2618. 33559097 10.1007/s11605-021-04913-6

[31] Frontali A Cohen L Bridoux V , et al. Segmental colectomy for ulcerative colitis: is there a place in selected patients without active colitis? An international multicentric retrospective study in 72 patients. J Crohns Colitis 2020; 14: 1687–1692. 32498084 10.1093/ecco-jcc/jjaa107

[32] Nakazawa T Naitoh I Hayashi K , et al. Inflammatory bowel disease of primary sclerosing cholangitis: a distinct entity? World J Gastroenterol 2014; 20: 3245–3254. 24696608 10.3748/wjg.v20.i12.3245 PMC3964396

[33] Gardenbroek TJ Pinkney TD Sahami S , et al. The ACCURE-trial: the effect of appendectomy on the clinical course of ulcerative colitis, a randomised international multicenter trial (NTR2883) and the ACCURE-UK trial: a randomised external pilot trial (ISRCTN56523019). BMC Surg 2015; 15: 30. 25887789 10.1186/s12893-015-0017-1 PMC4393565

