# Supplementary figures and images for: Clinical relevance of endoscopic peri-appendiceal red patch in ulcerative colitis patients
Source: Ther Adv Gastroenterol. 2022 Jun 28;15:17562848221098849. doi: 10.1177/17562848221098849 (PMC9244917; doi:10.1177/17562848221098849)

Supplementary file 1. Robarts Histopathology Index


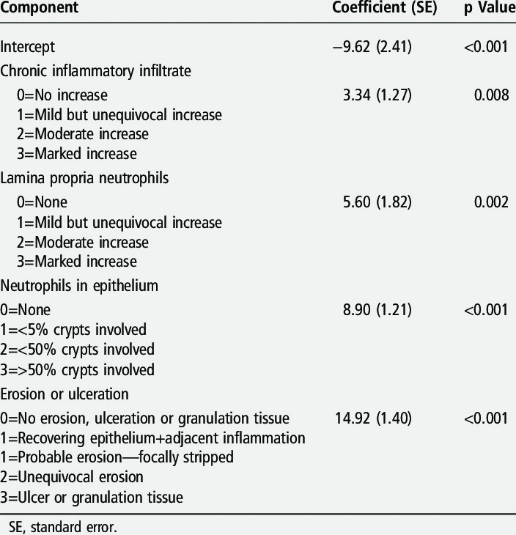

Supplement: sj-docx-1-tag-10.1177_17562848221098849 – Supplemental material for Clinical relevance of endoscopic peri-appendiceal red patch in ulcerative colitis patients [file sj-docx-1-tag-10.1177_17562848221098849.docx]
